# Supplementary figures and images for: Effect of Perinatal Ampicillin or Amoxicillin/Clavulanate Exposure on Maternal and Infant Gut Microbiome, Metabolome, and Infant Responses to the 20-valent Pneumococcal Conjugate Vaccine
Source: bioRxiv. 2025 Dec 8:2025.12.08.692990. Preprint. [Version 1] doi: 10.64898/2025.12.08.692990 (PMC12713748; doi:10.64898/2025.12.08.692990)

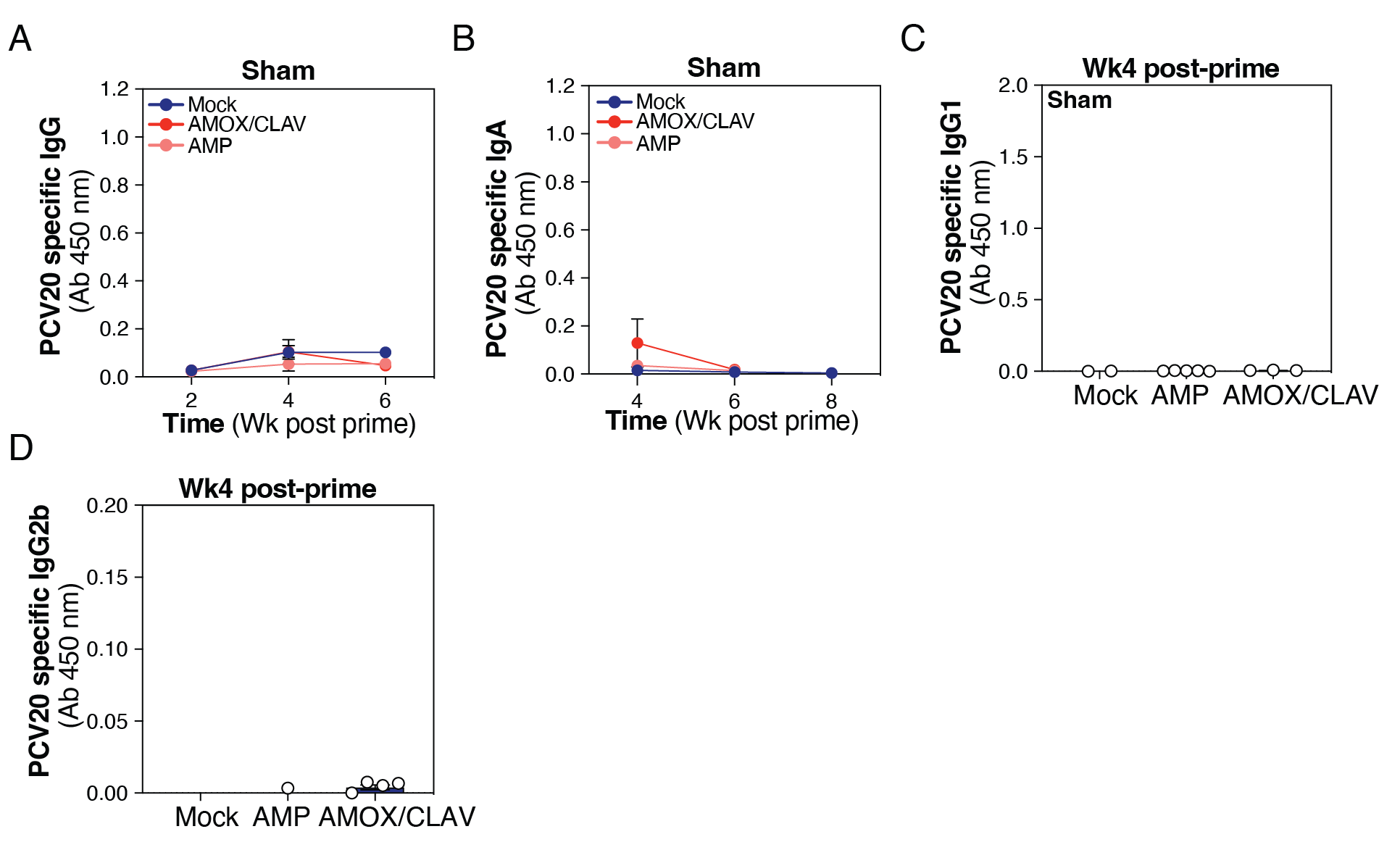

Supplement: Supplement 2 [file media-2.tif]

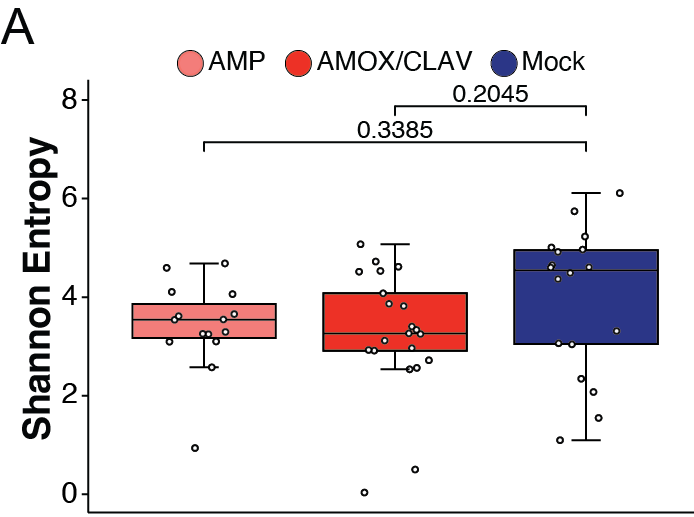

Supplement: Supplement 3 [file media-3.tif]

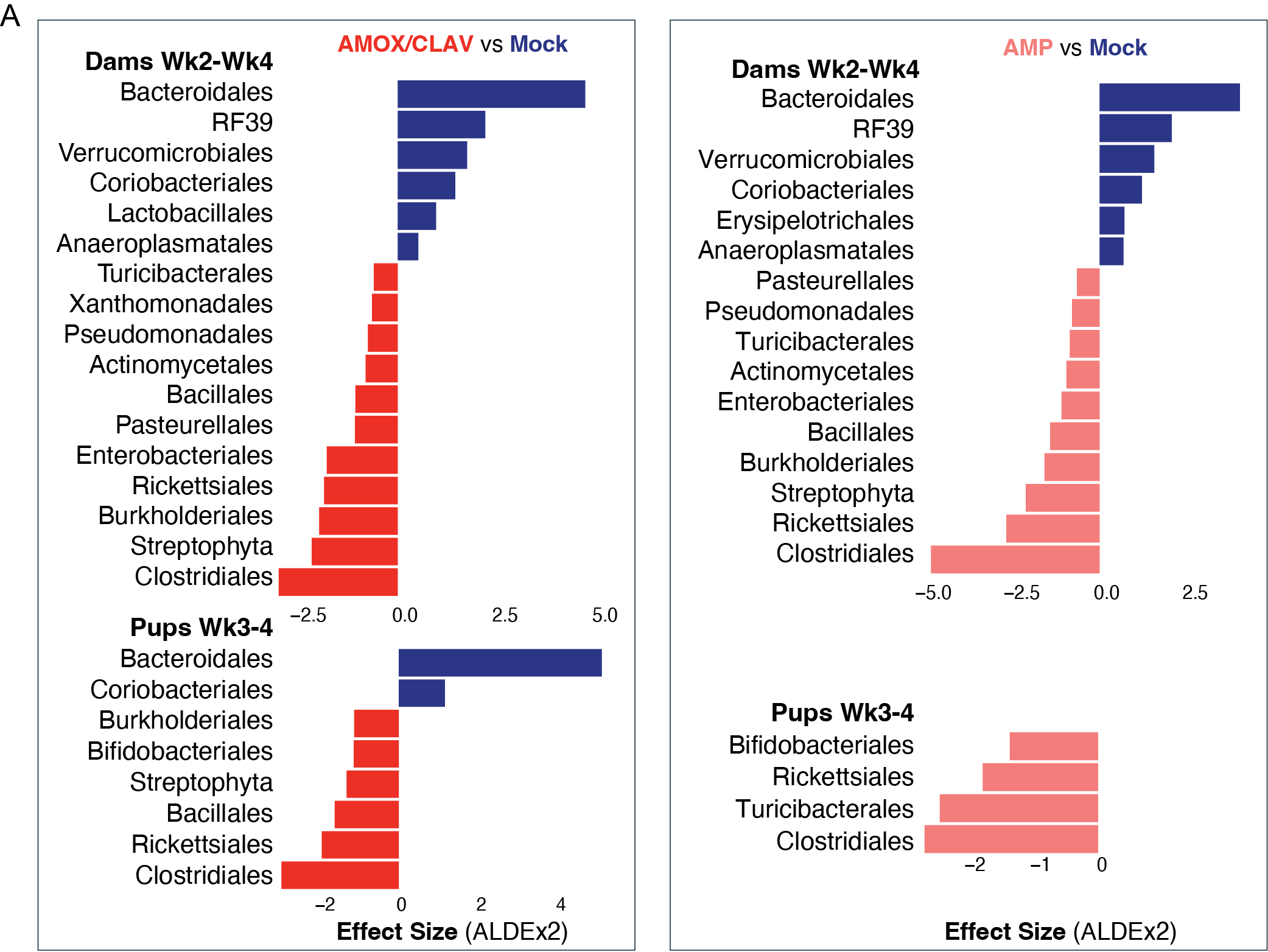

Supplement: Supplement 4 [file media-4.tif]

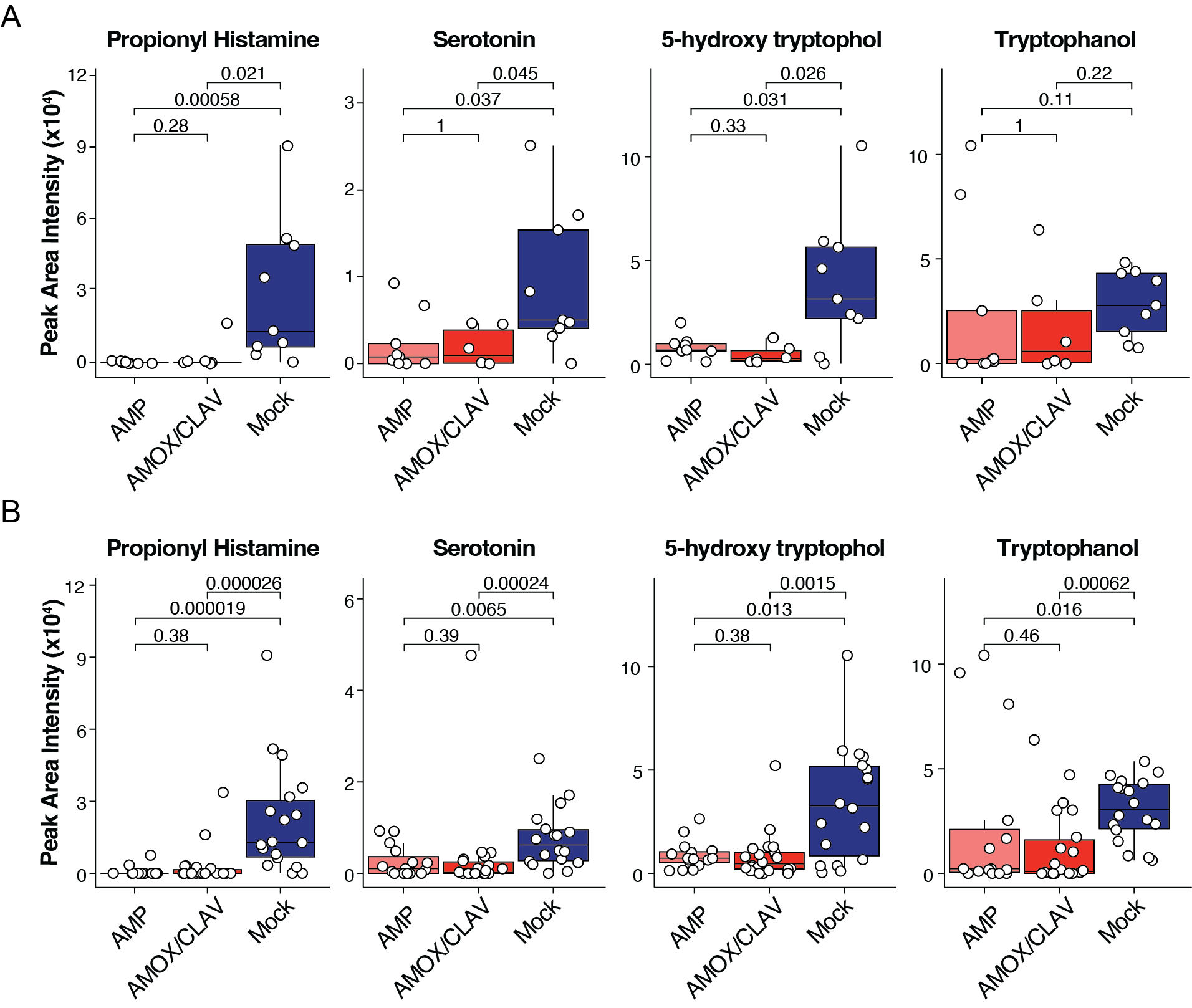

Supplement: Supplement 5 [file media-5.tif]

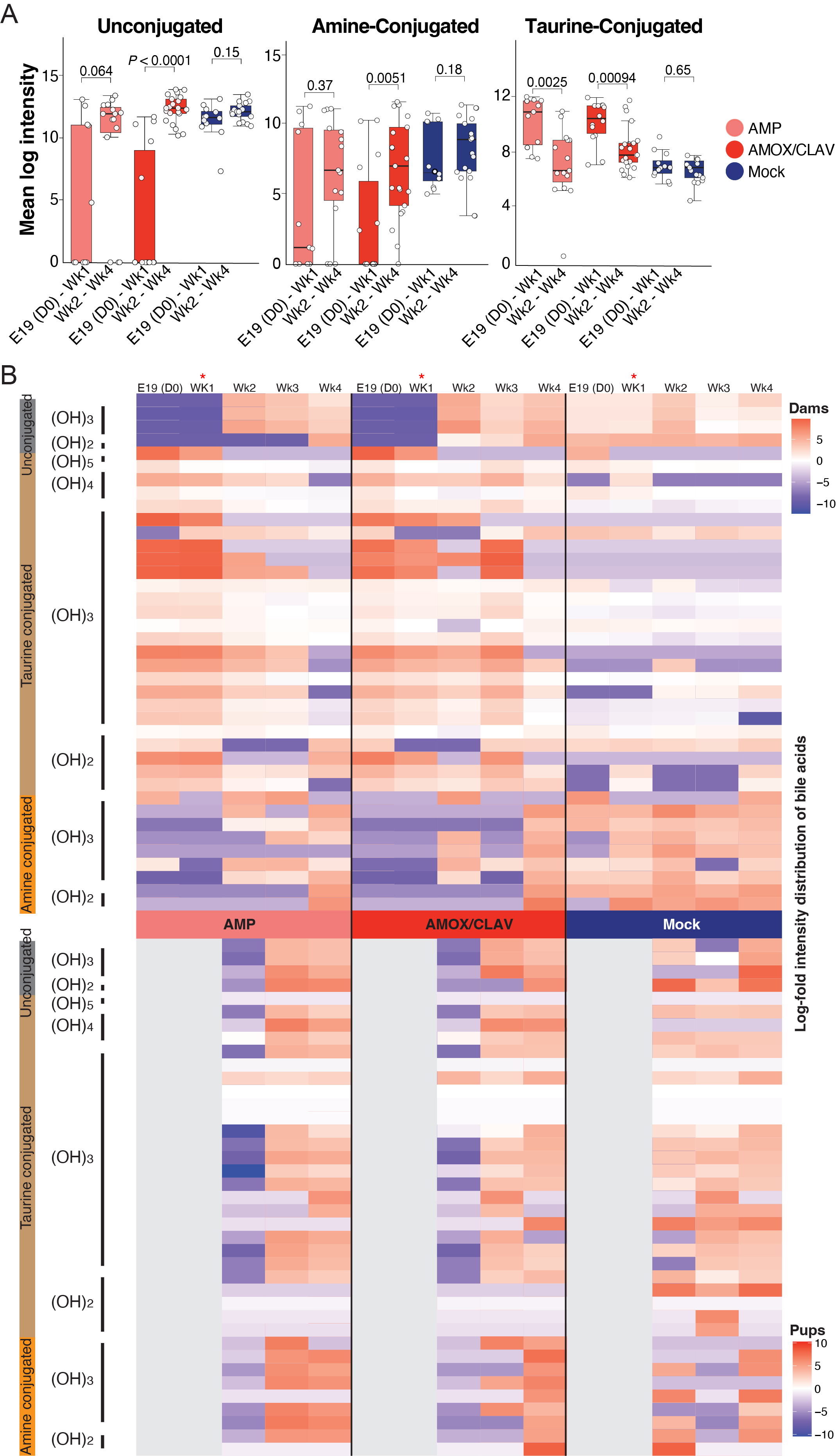

Supplement: Supplement 6 [file media-6.tif]

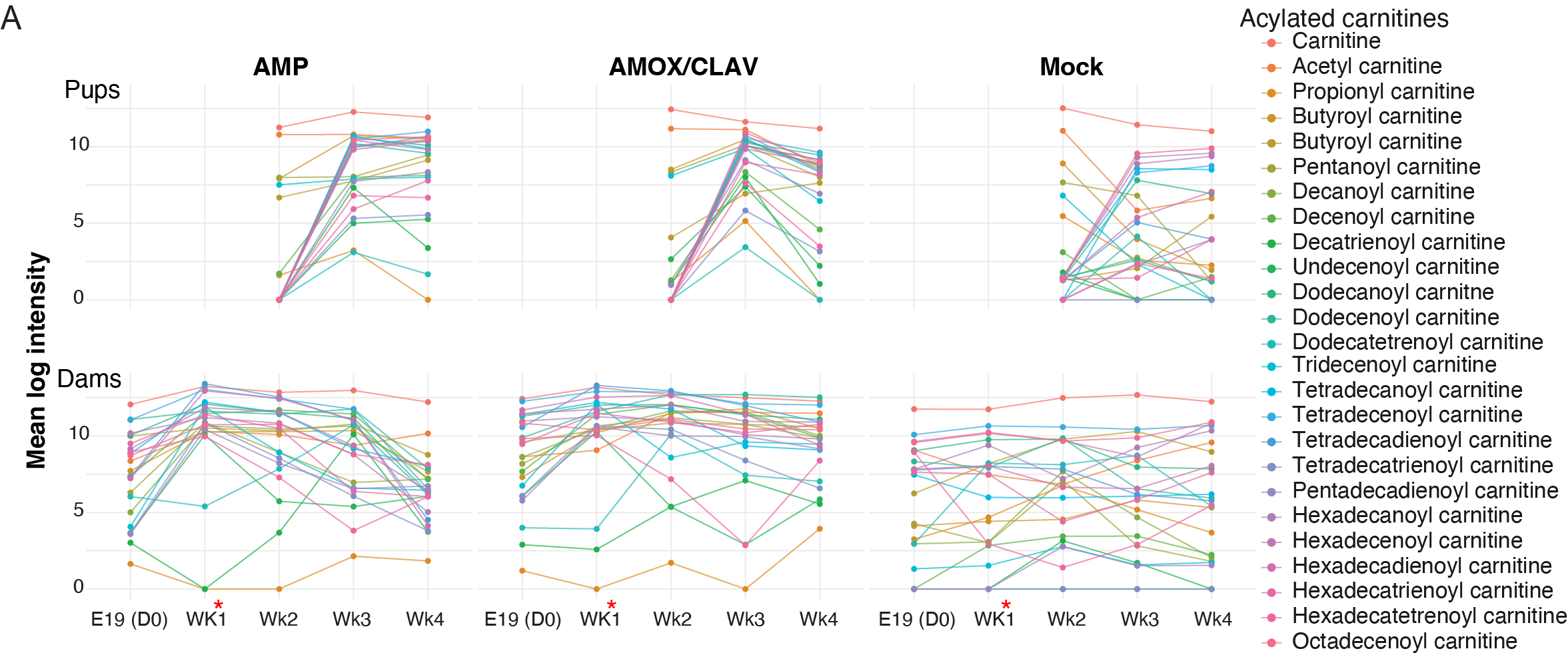

Supplement: Supplement 7 [file media-7.tif]

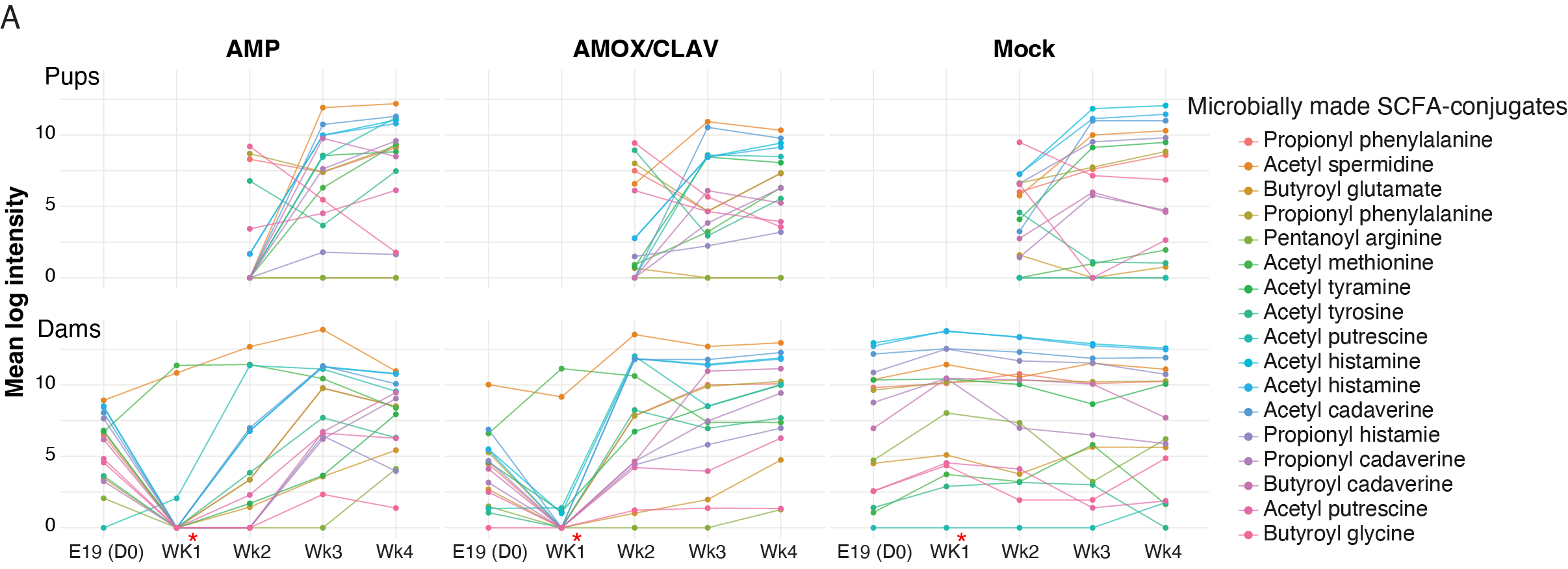

Supplement: Supplement 8 [file media-8.tif]
